# Plant-derived utility products: knowledge comparison across gender, age and education from a tribal landscape of western Himalaya

**DOI:** 10.1186/s13002-019-0346-8

**Published:** 2019-12-16

**Authors:** Alpy Sharma, Deepika Thakur, Sanjay Kr. Uniyal

**Affiliations:** High Altitude Biology Division, CSIR-Institute of Himalayan Bioresource Technology, Palampur, HP 176061 India

**Keywords:** *Bhangalis*, Bioresource, Himalaya, Tool, Traditional

## Abstract

**Background:**

Plant-derived utility products (PDPs) play an important role in sustaining humans, especially tribal communities. Despite this, knowledge on PDPs is declining.

**Methods:**

The present study was therefore carried to document the PDPs used by *Bhangalis,* a tribal community of western Himalaya, through door-to-door surveys (*n*=420) and detailed questionnaire recordings (*n*=240). In addition to taxonomic richness, frequency of species used; use value (UV), use diversity (UD), and cultural importance index (CI) were also analysed. Knowledge comparison between genders, four identified age groups (group 1: 16-32 years, group 2: 33–49 years, group 3: 50–66 years, group 4: >66 years), and level of education of the respondents was also carried out using multiple regression in SPSS.

**Results:**

*Bhangalis* reported 55 PDPs under five use categories namely tools (34), artefacts (7), construction and storage (6 each), and miscellaneous (2). For making these PDPs, 20 plant species representing 12 families were used. *Picea smithiana* (16.54%)*, Cedrus deodara* (14.96%)*, Cotoneaster bacillaris* (12.60%) and *Quercus semecarpifolia* (11.02%) reported the highest use frequency. On an average 15.13±0.25 PDPs per respondent was noted. Similarly, *Picea smithiana* (UV=0.088) and *Cedrus deodara* (UV= 0.079) reported the highest UV when compared to other species. Amongst all the species, maximum UD was revealed for *Juglans regia* in the tool category (0.91). On the other hand, maximum CI was also recorded for *Picea smithiana* (CI_Total_=2.91). With respect to gender, males were found to be more knowledgeable than females (*B*=6.370, *p*=0.001). Amongst the four age groups, respondents in group 1 (*B*=-13.302, *p*=0.001) and group 2 (*B*=-5.867, *p*=0.001) were less knowledgeable in comparison to respondents in the third and fourth age groups. Similarly, education also had a significant negative coefficient (*B*=-0.275, *p*=0.037), with educated respondents having lesser knowledge. It was observed that alternates available in the market seem to be limiting the use of PDPs.

**Conclusions:**

*Bhangalis* still use PDPs that have a crucial role in their lifestyle. However, their use is declining. A multipronged strategy that not only focusses on socio-economic characteristics but also on awareness especially at school levels is desired.

## Introduction

Himalaya, the youngest and the largest mountain range of the world is not only rich in biodiversity, but is also a home to many indigenous communities such as the *Gaddis*, *Gujjars*, *Apatanis*, *Garos*, *Mishmis, Bhangalis,* etc. [1]. These communities occupy different niches in the Himalaya and ever since their lifestyle have been guided by plants [2, 3]. As plants provided them, and still continue to provide them with a wide range of social and economic benefits, they are of immense importance and key to their livelihood [4]. From food to fibre, and medicine to shelter; all the major requirements of these communities are met from the forests [5–10]. With time they started processing the raw forest produce, i.e. wood, branches, logs, fibres, etc., for developing products such as tools, storage structures, artefacts, etc., that were useful in day-to-day life [9, 11, 12]. In Italy, Salerno et al. [5] noted the importance of plants in agriculture, domestic and handicraft sectors, and reported many novel uses of them. Importance of traditionally made storage structures in the life of ethnic communities has been highlighted by Sundaramari et al. [11]. At the same time, plant properties in relation to making products have also been emphasized [13]. Recently, Kang et al. [10] presented information on plants as precursors of various products in China. Further, studies on the subject have argued that traditional products are environment friendly and can be used in designing modern day equipment [14]. Thus, it is evident that tribal communities have vast knowledge on the utilization of plants that they have gained over time through trial and error. However, this knowledge is fast declining [15, 16] and as oral transmission of traditional knowledge from older to younger generation is not always assured [17, 18], documentation of this knowledge becomes important [19–22].

*Bhangalis* represent a highly knowledgeable tribe of the western Himalaya that uses plant resources in its daily chores. Their knowledge on use of plants as medicines [7] and that for edible purposes is well recognized with many new uses that were hitherto unknown [23]. Farming and animal husbandry is the mainstay of *Bhangalis* for which they use the surrounding resources. Unfortunately, their knowledge on PDPs that meet their requirements of farming and animal husbandry largely remains undocumented.

Documentation of this knowledge becomes all the more important as recent studies have noted a change in agricultural pattern, declining use of bioresources, and a trend of depleting traditional knowledge in the western Himalaya [24, 25]. The knowledge and practices of tribal people in addition to cultural factors is also influenced by socio-economics [26–30]. Recognizing this, the present study was undertaken to: (1) document indigenous PDPs used by the *Bhangalis*, (2) identify the species used for making PDPs, and (3) compare knowledge differences with respect to gender, age and education. We hypothesized that sociocultural factors are important in shaping the knowledge.

## Materials and methods

### Study area

The study was conducted in Chhota Bhangal area of western Himalaya that lies at coordinates 32°04’32.83” N and 76°51’30.45” E in the lap of Dhauladhar mountain range. The area is drained by Uhl and Lambadug rivulets, the catchments of which are formed by the temperate Himalayan forests comprising oaks and conifers that are rich in medicinal plants [31]. The Shanan Hydro-Electric Project built on Uhl in 1930 is amongst the pioneer hydel power project of north India. Sandstones, silt stone, phyllite and quartzite characterize the rock types of the valley [32]. The area receives heavy snowfall during winters (December–January) while July–August are the months of heavy rainfall. The temperature ranges from a minimum of -10° C during January to a maximum of 34° C in June [33]. In addition to being popular as an adventure tourism site, the area is known for trout fish.

The residents of the area are referred to as *Bhangalis* with agriculture being their main occupation. Barley, maize, and rajmah are the major cereal and legume crops grown by them while potato, radish, cabbage, cauliflower and tomato are the common vegetables grown in the area. Apart from agriculture, they rear livestock for milk and draught power. Sheep and goat are kept for meat and wool. *Bhangalis* have a rich legacy of using natural resources in their day-to-day life [7, 23] and thus are a storehouse of traditional knowledge.

### Field surveys

The study involved regular field surveys to Chhota Bhangal, and between August 2016 and September 2018 a total of seven surveys ranging from a minimum of five days to a maximum of 20 days were conducted to the area. Initial reconnaissance surveys coupled with our background work in the area [7, 23, 31] helped in identification of six villages for detailed investigations (Table 1).

**Table 1:** Locational characteristics of the studied villages

S. no.	Name of village	Latitude	Longitude	Altitude (m)
1.	Termehr	32°04’28.606”	76°51’19.858”	2100
2.	Judhar	32° 04’42.06”	76° 50’50.001”	2450
3.	Bhujling	32°06’03.73”	76°51’14.880”	2180
4.	Punag	32°05’35.753”	76°51’20.954”	2230
5.	Andarli Malahn	32°04’24.762”	76°52’01.67”	2200
6.	Lwai	32°03'29.632”	76°51'22.792”	2018

Rapid door-to-door surveys covering all the households (*n*=420) in the six villages were conducted. Also, free listing of species and PDPs was carried out using PRA [34, 35]. Here emphasis was on generating primary data on age, education, and profession of the resident population (Additional file 1: Table S1). This guided stratified random selection of respondents for detailed statistical knowledge comparisons (*n*=240) between respondents belonging to different age groups, and gender [10]. The information was collected anonymously. Later, walks in the wild were organized with the local people so as to collect specimens of the species used for making the PDPs using standard methods [36]. All the collected specimens are housed in the herbarium (PLP) of the CSIR-Institute of Himalayan Bioresource Technology, Palampur.

As mandated by National Biodiversity Authority, oral prior informed consent of all the informants was obtained.

#### Analyses and ethnobotanical indices

The data were analysed for PDPs used. Based on their use, the same have been classified into five categories namely tools, storage structures, construction use, artefacts, and miscellaneous (Table 2). Analyses of taxonomic richness, frequency and percentage use of species for making PDPs has been done [10]. Data collected were also analysed for use value (UV), use diversity (UD), and cultural importance index (CI).

*Use value:* the UV helps in determining as to which species is most frequently used by the community. It was calculated using the following equation:

**Table 2:** Categorization of products and their description

S. no.	Categories	Description
1	Tools	Products designed and used for performing specialized tasks or activities
2	Storage structures	Products used for storing grains or other household items
3	Construction use	Products that are shelter oriented and used for construction
4	Artefacts and handicraft	Items made by bare hand for decorative purpose or daily use
5	Miscellaneous	This category includes only two products, one for supporting creepers in agricultural fields, and the other for sitting

UV= **Ʃ***U*_is_/ *n* where *U*_is_ is the number of uses mentioned by the informant ‘i’ for a given species ‘s’, and ‘*n*’ is the total number of respondents [37].

*Use diversity*: it provides an aggregate of different use categories in which a species is used and how they contribute to the cumulative use of species. In our case five use categories were identified (tools, artefacts and handicrafts, storage structures, construction, and miscellaneous). UD was calculated using the formula- UDV= *U*cx/*U*ct, where *U*cx is the number of indications recorded of a species in a category and *U*_ct_ is the total number of indications for all categories [26].

*Cultural importance index* The cultural significance of species was assessed through CI and was calculated using the given formula:

$$ {\mathrm{CI}}_{\mathrm{S}}={\sum}_{u-u1}^{uNC}\sum \limits_{i=i1}^{iN}\frac{URui}{N} $$

It is the sum of use report (UR) in each PDP category mentioned for a species divided by the number of participants (*N*) [38].

### Knowledge comparison

To study the knowledge variations between respondents of different gender, age groups and education levels, multiple regression analyses were carried out in SPSS. Statistical model was used to explore how the above three sociocultural variables relate to knowledge about PDPs. Gender had two categories (male and female), age was categorized into four groups (group 1: 16–32 years, group 2: 33–49 years, group 3: 50–66 years, group 4: >66 years) while education (0–17) was treated as a continuous variable (Additional file 1: Table S1). During analyses, these variables were treated as independent variables and the categorical variables were coded for analysis. Male was coded as 1, female as 0 while for the age groups dummy variables D1, D2, D3 and D4 were used. We considered *p* values < 0.05 as statistically significant [39, 40]**.**

## Results

### Plant-derived products

**Fig. 1:**
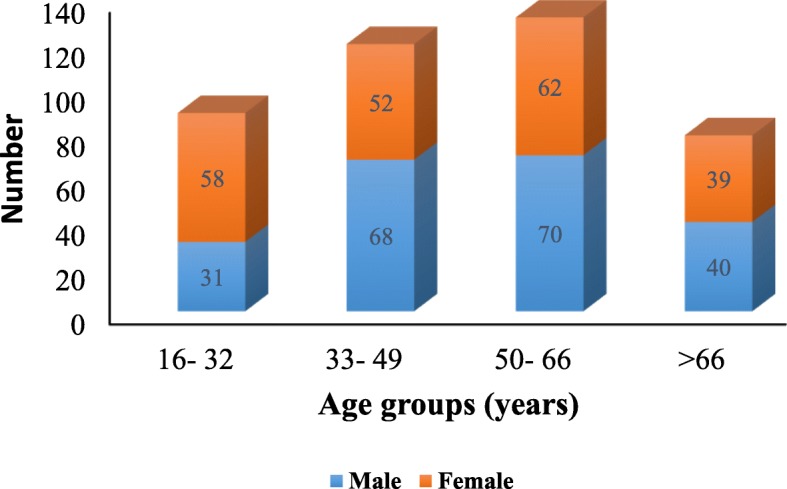
Age and gender characteristics of the respondents

**Fig. 2:**
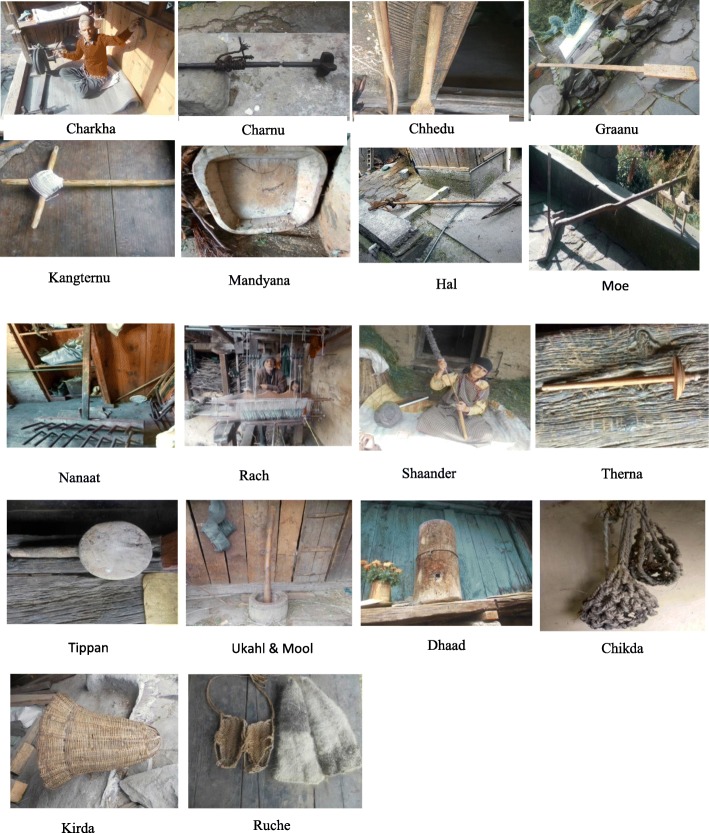
Field photographs of the products used by the *Bhangalis*

A total of 420 individuals comprising males and females in different age groups were surveyed through door-to-door household interactions in a participatory mode (Fig 1). Majority of these reported having agriculture as their main profession (~97%) while only ~29% reported having received formal education. Thus, more than 70% of the respondents did not have formal education (Additional file 1: Table S1). Compilation of free lists revealed use of 20 species for making 55 PDPs by the *Bhangalis* (Fig 2). The 55 PPDs used by the *Bhangalis* can be classified into five major types:

#### Tools

Out of the 55 PDPs, 34 (61.82% of total PDPs) were used as tools for carrying out specific tasks and activities. From being used to carrying loads, spinning fibres, cooking food, to removing snow; tools find multiple utility. Characteristics of each of these tools are provided in Table 3.

**Table 3:** Products, their characteristics, uses and the species used for making them

S. no.	Product	Uses	Species used
Tools			
1.	Basola	For shaping wooden implements and other artefacts	*Cotoneaster bacillaris, Quercus semecarpifolia*
2	Bralu	For collection of leaf litter and for the seperation of husk during grain threshing	*Cotoneaster bacillaris, Sorbaria tomentosa*
3	Buhaar	For cleaning house and the surroundings	*Arundinaria falcata, Pinus wallichiana, Spiraea canescens*
4	Charkha	For spinning natural fibres into thread	*Juglans regia*
5	Charnu	For churning fresh homemade curd	*Juglans regia, Picea smithiana, Quercus semecarpifolia*
6	Chhedu	For cooking feasts in temple	*Cotoneaster bacillaris*
7	Chosar	For cleaning and sanding surfaces	*Cotoneaster bacillaris, Juglans regia*
8	Daangu	For support while walking	*Cotoneaster bacillaris, Quercus semecarpifolia, Sorbaria tomentosa, Viburnum erubescens*
9	Dbotan	For washing cloths	*Cedrus deodara, Picea smithiana*
10	Draati	For harvesting crops or forage	*Cotoneaster bacillaris, Quercus semecarpifolia, Salix alba*
11	Fauda	For collecting grains & also cow dung	*Cedrus deodara, Cotoneaster bacillaris, Picea smithiana*
12	Graanu	For removing snow	*Abies pindrow, Cedrus deodara, Picea smithiana, Pinus wallichiana*
13	Hal	For ploughing fields	*Quercus semecarpifolia, Salix alba, Taxus baccata*
14	Hathoda	For fixing nails and breaking apart objects	*Cotoneaster bacillaris, Quercus semecarpifolia*
15	Julnu	For carrying fodder	*Cedrus deodara, Picea smithiana*
16	Kangternu	For making ropes	*Cotoneaster bacillaris*, *Sorbaria tomentosa, Spiraea canescens*
17	Khis	For making marks on wood	*Cotoneaster bacillaris, Juglans regia*
18	Kudali	For digging and weeding	*Cotoneaster bacillaris, Quercus semecarpifolia*
19	Kulhadi	For shaping, splitting and cutting wood	*Cotoneaster bacillaris, Quercus semecarpifolia*
20	Mandyana	For thickening *pattu* (hand weaved cloth)	*Cedrus deodara, Picea smithiana, Juglans regia*
21	Mattiyaan	For breaking clods	*Cotoneaster bacillaris, Quercus semecarpifolia*
22	Moe	For levelling the land after ploughing	*Quercus semecarpifolia*
23	Naalu	For directing air to boost wooden fire	*Arundinaria falcata*
24	Nanaat	For arranging threads before weaving	*Cedrus deodara*, *Picea smithiana*
25	Nihaan	For making wooden items. It is used to fix the window and door	*Cotoneaster bacillaris, Juglans regia*
26	Pithu	For carrying stones or bricks on back	*Aesculus indica, Juglans regia, Picea smithiana, Ulmus wallichiana*
27	Rach	For weaving woollen items	*Arundinaria falcata, Juglans regia, Ulmus wallichiana*, *Picea smithiana*, *Cedrus deodara*
28	Randa	For smoothening wood	*Cotoneaster bacillaris, Juglans regia*
29	Shaander	For pre-processing of fibre before weaving	*Arundinaria falcata*
30	Therna	For making yarn from wool	*Quercus semecarpifolia, Sorbaria tomentosa*
31	Tippan	For crushing fruits of *Aesculus indica* to make flour	*Juglans regia*, *Quercus semecarpifolia, Taxus baccata, Ulmus wallichiana*
32	Toka	Wooden implement upon which wood or meat is cut	*Quercus semecarpifolia*
33	Trethu	For hand threshing of cereals	*Cotoneaster bacillaris*
34	Ukhal and Mool	For grinding and milling	*Cedrus deodara*, *Picea smithiana, Quercus semecarpifolia*, *Taxus baccata, Ulmus wallichiana*
Storage structures			
35	Bhaad	For storing dried fodder	*Cedrus deodara, Picea smithiana*
36	Bhujnu	For storing fodder in an open place	*Cedrus deodara, Picea smithiana*
37	Dhaad	For rearing honey bees	*Ulmus wallichiana*
38	Kothar	For keeping daily use items such as ration or cloths	*Abies pindrow*, *Cedrus deodara, Picea smithiana*
39	Mlaii	For storage of cow dung as manure	*Cedrus deodara, Picea smithiana,*
40	Pedu	For storing crops such as potato	*Arundinaria falcata,Ulmus wallichiana*
Construction use			
41	Baada	A gate restricting entry of animals into house	*Cedrus deodara, Picea smithiana*
42	Ghar	House for living	*Cedrus deodara, Picea smithiana, Abies pindrow*, *Pinus wallichiana*
43	Mandar	For worshipping deities	*Cedrus deodara, Picea smithiana*
44	Oda	For keeping hens	*Cedrus deodara, Picea smithiana, Pinus wallichiana, Ulmus wallichiana*
45	Puliya	For crossing small rivulets	*Cedrus deodara, Picea smithiana*
46	Siddi	For climbing to reach higher places	*Cedrus deodara, Picea smithiana*
Artefacts			
47	Chikda	For covering mouth of animals to prevent grazing	*Cannabis sativa*
48	Kirda	For carrying cow dung and other farm products	*Arundinaria falcata*
49	Mandari	Mat for sitting or lying	*Hordeum vulgare, Triticum aestivum*
50	Rassi	For tieing animals, fodder and other artefacts	*Cannabis sativa*
51	Ruche	Shoes for walking over snow	*Cannabis sativa*
52	Traani	For drying household use material	*Arundinaria falcata*
53	Treda	For carrying objects on head	*Hordeum vulgare, Triticum aestivum*
Miscellaneous			
54	Jyun	For supporting creepers/vines such as Rajmah, Cucumber, etc.	*Arundinaria falcata, Desmodium elegans, Indigofera heterantha*, *Sorbaria tomentosa*
55	Patdu	Stool for sitting	*Cedrus deodara*, *Juglans regia*, *Picea smithiana*, *Pinus wallichiana*

#### Storage

Six PDPs that amounts to ~11% of the total PDPs represent storage structures that were used for storing grains and other household items such as utensils, cloths, etc. (Table 3).

#### Construction

These are used for building structures and alike storage structures, six PDPs (10.91%) fall under the construction category. These include *Baada, Ghar, Mandar*, *Oda, Puliya* and *Seedi* (Table 3).

#### Artefacts and handicrafts

*Bhangalis* are experienced in making various artefacts and handicrafts. Seven PDPs (12.73%) fall under this category that includes *Ruche* and *Chikda*. While culms of *A. falcata* were used for making *Kirda* and *Traani*, people made *Mandari* and *Treda* using straws of *Triticum aestivum* and *Hordeum vulgare* (Table 3).

#### Miscellaneous

Two PDPs namely *Jyun* and *Patdu* fall under this category. *Jyun* is used for supporting vines and climbers, and mainly comprise *Arundinaria falcata* culms. *Patdu*, on the other hand is rectangular stool used for sitting (Table 3).

### Species used

**Fig. 3:**
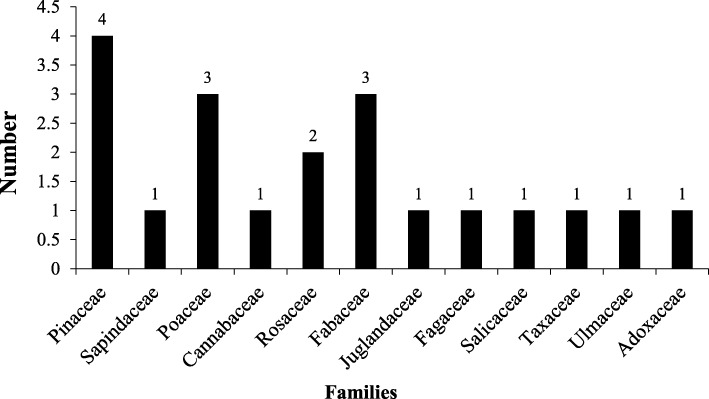
Statistics of family to which the species used belong

**Fig. 4:**
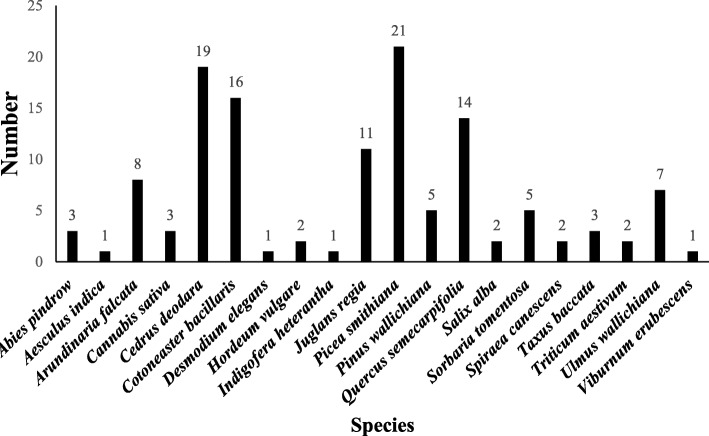
Frequency of the species use

Twenty plant species belonging to 12 families were reported by the *Bhangalis* for making the 55 PDPs (Table 4). Through walks in the wild, specimens of all these species have been collected and are accessioned in the PLP herbarium. Maximum number of the species belong to the family Pinaceae (4) followed by Poaceae, Fabaceae (3 each), and Rosaceae (2). The remaining 8 families were represented by 1 species, each (Fig 3). With respect to life form, maximum of these are tree (50%), followed by shrub (30%), grass (15%), and herb (5%) (Table 4). Amongst the species used, frequency of use of *Picea smithiana* was the highest (16.54%) that was followed by *Cedrus deodara* (14.96%)*, Cotoneaster bacillaris* (12.60%) and *Quercus semecarpifolia* (11.02%) (Fig 4 ).

**Table 4:** Characteristics of the species used and a comparative account of their uses with other studies

S. no.	Species (family) collection number	Local name	Life form	Wood characteristics (based on [41, 42])	Present study	Other studies
I	*Abies pindrow* Royle (Pinaceae) PLP 9977	Tosh	Tree	It is soft and easy to saw wood, the weight of which is about 30 lbs per cubic foot	Tool, storage structure, construction	House construction [43–47]; household articles, furniture [46].
2	*Aesculus indica* Hook (Sapindaceae) PLP 9927	Khnor	Tree	The wood is soft white that polishes well and weighs 34 lbs per cubic foot.	Tool	Palanquins [43]; household articles, furniture [46]; agricultural implements, yoke, hoe [45, 47, 48].
3	*Arundinaria falcata* Nees. (Poaceae) PLP 9978	Nagaal	Grass (bamboo)	A multipurpose bamboo. Its strength and light weight render it suitable for making products.	Tool, storage structure, artefact, miscellaneous	Household articles, baskets, mat, hat, broom, and winnow [43]
4	*Cannabis sativa* L*.* (Cannabaceae) PLP 9945	Bhangolu	Herb	A fibre yielding plant. It produces more fibre than cotton and flex	Artefacts	Ropes [43], basket and mat [49]
5	*Cedrus deodara* G. Don (Pinaceae) PLP 9979	Deodar	Tree	Strongest amongst Indian conifers, it is easy to saw and work. Its weight is about 35 lbs per cubic foot	Tool, storage structure, construction, miscellaneous	Furniture, house construction, door, windows, carvings [43–45, 47.
6	*Cotoneaster bacillaris* Wall. ex Lindl. (Rosaceae) PLP 9980	Riunsh	Shrub	The wood is hard, close and even grained. Its weight is about 57 lbs	Tools	Agricultural tools, implements [43, 48, 50]
7	*Desmodium elegans* DC. (Fabaceae) PLP 9981	Safed kathi	Shrub	A common shrub of the Himalayan region	Miscellaneous	Ropes, sacs [45], tools [48]
8	*Hordeum vulgare* L. (Poaceae) PLP 9982	Joo	Grass	Surface roughness and polarity of its fibre are of importance	Artefact	-
9	*Indigofera heterantha* Baker (Fabaceae) PLP 9983	Kai kathi	Shrub	The wood is hard and weighs around 55 lbs per cubic foot	Miscellaneous	Tool, handles of axe, pick axe, scythe, hammer [48]
10	*Juglans regia* L. (Juglandaceae) PLP 9959	Khod	Tree	The wood is light, durable and has good working qualities. Its weight is about 44 lbs per cubic foot	Tool, miscellaneous	Construction, furniture, cabinets [43, 44]; agricultural implements, plough, yoke [46–48].
11	*Picea smithiana* Boiss. (Pinaceae) PLP 9984	Rai	Tree	Light weight, it is easy to saw and work. Its weight is about 31lbs per cubic foot	Tool, miscellaneous, storage structure, construction	Storage structures, boxes [47]; house construction [44].
12	*Pinus wallichiana* A. B. Jacks. (Pinaceae) PLP 9985	Kail	Tree	Wood is fairly durable and of good quality. Its weight is about 32 lbs per cubic foot	Tool, construction, miscellaneous	Furniture [43]; house construction, door, windows, shutter [44, 47]; tools [51].
13	*Quercus semecarpifolia* Sm. (Fagaceae) PLP 9986	Khreu	Tree	Its weight is about 54 lbs per cubic foot and is used on account of its strength and durability	Tool	House construction [43, 47], agricultural implements, ploughs [43, 46, 48]; furniture, bed, table [49].
14	*Salix alba* L. (Salicaceae) PLP 9987	Bhasal	Tree	It is a lightwood that weighs about 25 lbs per cubic foot	Tool	-
15	*Sorbaria tomentosa* Rehder (Rosaceae) PLP 9988	Kust	Shrub	The wood is hard and compact	Tool, miscellaneous	-
16	*Spiraea canescens* D. Don. (Fabaceae) PLP 9989	Chakhu	Shrub	Wood is fairly hard and even grained	Tool	Agricultural implements, tool [50]
17	*Taxus baccata* L. (Taxaceae) PLP 9948	Rakhal	Tree	Strong wood that polishes beautifully. Its weight is 44 lbs per cubic foot	Tool	Vases, Pots, Containers , Palanquins [43], Construction, [44–46]
18	*Triticum aestivum* L. (Poaceae) PLP 9992	Gehu	Grass	Household cereal crop. Fibre used due to its low cost and environmental friendly nature	Artefacts	Craft for decoration [48]
19	*Ulmus wallichiana* Planch. (Ulmaceae) PLP 9990	Maraal	Tree	Wood is fairly hard, scented and fine grained. Its weight is about 36 lbs per cubic foot	Tool, storage structure, construction	Miscellaneous [43]
20	*Viburnum erubescens* Wall. (Adoxaceae) PLP 9991	Talyana	Shrub	Common Himalayan shrub.	Tool	-

### Ethnobotanical indices

#### Use value

For different species, the UV ranged form 0.004 to 0.088. *Picea smitiana* (0.088), *C. deodara* (0.079) and *C. bacillaris* (0.067) reported higher UV. They were followed by *Q. semecarpifolia* (0.058), *Juglans regia* (0.046), and *A. falcata* (0.033) and *Ulmus wallichiana* (0.029). *Desmodium elegans* and *Indigofera heterantha* (0.004, each) reported the lowest UV (Table 5).

**Table 5:** Use value, use diversity and cultural importance index of the species used for making plant derived products

Species	UV	UD_T_	UD_S_	UD_C_	UD_A_	UD_M_	CI_T_	CI_S_	CI_C_	CI_A_	CI_M_	CI_Total_
*Abies pindrow*	0.013	0.50	0.50	-	-	-	0.01	0.19	0.11	-	-	0.31
*Aesculus indica*	0.004	-	-	-	-	-	0.05	-	-	-	-	0.05
*Arundinaria falcata*	0.033	0.50	0.13		0.25	0.13	0.83	0.08	-	0.93	0.25	2.1
*Cannabis sativa*	0.013	-	-	-	-	-	-	-	-	0.91	-	0.91
*Cedrus deodara*	0.079	0.42	0.21	0.32		0.05	0.46	0.46	0.81	-	0.03	1.76
*Cotoneaster bacillaris*	0.067	-	-	-	-	-	1.30	-	-	-	-	1.30
*Desmodium elegans*	0.004	-	-	-	-	-	-	-	-	-	0.35	0.35
*Hordeum vulgare*	0.008	-	-	-	-	-	-	-	-	0.55	-	0.55
*Indigofera heterantha*	0.004	-	-	-	-	-	-	-	-	-	0.35	0.35
*Juglans regia*	0.046	0.91	-	-	-	0.09	0.79	-	-	-	0.01	0.80
*Picea smithiana*	0.088	0.48	0.19	0.29		0.05	0.72	0.68	0.93	-	0.58	2.91
*Pinus wallichiana*	0.021	0.40		0.40	-	0.20	0.004		0.021	-	0.029	0.05
*Quercus semecarpifolia*	0.058	-	-	-	-	-	0.72	-	-	-	-	0.72
*Salix alba*	0.008	-	-	-	-	-	0.16	-	-	-	-	0.16
*Sorbaria tomentosa*	0.021	0.80	-	-	-	0.20	0.60	-	-	-	0.70	1.30
*Spiraea canescens*	0.008	-	-	-	-	-	0.24	-	-	-	-	0.24
*Taxus baccata*	0.013	-	-	-	-	-	0.19	-	-	-	-	0.19
*Triticum aestivum*	0.008	-	-	-	-	-		-	-	0.38	-	0.38
*Ulmus wallichiana*	0.029	0.57	0.29	0.14	-	-	0.10	0.30	0.01	-	-	0.41
*Viburnum erubescens*	0.004	-	--	-	-	-	0.01	-	-	-	-	0.01

UV= use value, UD= use diversity value, UD_T_= use diversity value for the tool, UD_S=_ use diversity value for the storage structure, UD_C=_ use diversity value for the construction category, UD_A=_ use diversity value for the artefact category, UD_M=_ use diversity value for the miscellaneous category, CI= cultural importance index, CI_T_= cultural importance for tool category, CI_S_= cultural importance for the category of storage structure, CI_C_= cultural importance for the category construction, CI_A_= cultural importance for the category artefacts, CI_M_= cultural importance for the miscellaneous category.

#### Use diversity

In terms of use of different species for the five identified use categories of PDPs, most of the interviewees mentioned tools. The use of tools is most diversified and most of plant species were used for making them. In tools, maximum UD, i.e. 0.91 was recorded for *J. regia* (Table 5).

#### Cultural importance index

*Picea smithiana* (CI_Total_ = 2.91) followed by *A. falcata* (CI_Total_ = 2.1) and *C. deodara* (CI_Total_ = 1.76) reported the overall highest CI while the lowest was reported by *Viburnum erubescens* (CI_Total_ = 0.01). With reference to different use categories, *C. bacillaris* reported maximum CI in the tool category (CI_T_= 1.30). For storage structure and construction categories (CI_S_= 0.68, CI_C_= 0.93; respectively) maximum CI values were reported for *P. smithiana* while for the miscellaneous category maximum CI value was recorded for *Sorbaria tomentosa* (CI_M_ = 0.70). In case of artefacts, *A. falcata* had the highest CI value, i.e. 0.93 (Table 5).

### Knowledge comparison

On an average, 15.13±0.25 PDPs per respondent were listed. However, for males the average number of PDPs per respondent was 16.85±0.36 while for the females the same was 13.42±0.28. Statistically, we found a significant positive cofficient for the variable gender (*B*= 6.370, *p*=0.001) wherein males were more knowledgeable in comparison to females (Table 6). With respect to different age groups, average number of PDPs per respondent for the first age group was 12.37±0.43, while for the fourth age group the same was much higher, i.e. 16.29±0.58. On comparing different age groups, we found that the first (16–32) and second age group (33–49) respondents significantly negatively correlated with knowledge while in the case of third age group (50–66) no significant difference was found. The results show that respondents in the first (*B*=-13.302, *p*= 0.001) and second age groups (*B*=-5.867, *p*= 0.001) posess lesser knowledge on species used for making PDPs (Table 6).

**Table 6:** Knowledge of plant species used for making PDPs in relation to age, gender and education

Explanatory variables	Factors	Values
Gender (categorical)	Male (120)	*B*= 6.370, *p*= 0.001
	Female (120)	
Age (categorical)	D1 (54)	*B*= -13.302, *p*= 0.001
	D2 (59)	*B*= -5.867, *p*= 0.001
	D3 (78)	*B*= -0.369, *p*= 0.784
	D4 (49)	
Education (continuous)	240	*B*= -0.275, *p*= 0.037

Similarly, education was significantly negatively correlated to species knowledge. With literacy, the knowledge about the species used for making PDPs declined (*B*=-0.275, *p*= 0.037) (Table 6).

## Discussion

History of human evolution has revolved around natural resources and their use for multifarous applications [2, 52]. The *Bhangalis* of western Himalaya not only use plants as medicine [7] and food [23], but also for developing daily use household products. Despite richness of species in their surroundings, they only used 20 species for making an array of products. This could be attributed to the fact that these species are the dominant species of the western Himalayan region and are easily available in their vicinity [53, 54]. This probably minimizes their collection effort and time. Reports of use of commonly occurring species in routine life are avaiable from many regions of the globe where minimization of effort and maximization of output has been emphasized [5, 10, 55, 56].

### PDPs and species used

Amongst these 20 species, the frequency of use of *P. smithiana* (16.54%)*, C. deodara* (14.96%)*, C. bacillaris* (12.60%) and *Q. semecarpifolia* (11.02%) was the highest. These species also reported higher UV, UD and CI values (Table 5) thereby indicating their importance to the local people. This may be because temperate Himalayan forests comprising *C. deodara, P. smithiana*, and *Q. semecarpifolia* dominate the surrounding landscape of *Bhangalis*. *Cotoneaster bacillaris* is considered highly robust and therefore may be more used [33, 53]. In addition to being common, use of certain species can be linked to their unique properties. *Cedrus doadara* that was used for construction products is known for its durability and resistance to pests [57]. It is the strongest of Indian conifers and thus is suited for structural and building works [58, 59]. In addition to its durability, *Bhangalis* consider *C. deodara* as water resistant and therefore products that often come in contact with water such as *mandyana* and *patdu* were made up of its wood. In industry also, *C. deodara* is highly preferred for making furniture and in construction purposes [46]. *Picea smithiana*, on the other hand, is light weight and therefore products made from it are easy to carry and use. Elsewhere also, tribal communities refer to it as a light wood species [60]. The other frequently used species was *Q. semecarpifolia*. *Bhangalis* mentioned it to be a strong wood and recommended it for making ploughs and other products. In other Himalayan areas also, *Quercus* is used for making plough due to its strength [48, 61]. Gamble [41] reported that the strength and durability of oaks is very high. *Bhangalis* specifically pointed to using *S. alba* for making neck yoke for oxen, which is used for plouging. According to them, it is light in weight and thus can be carried on neck for longer durations [62]. Further, *Bhangalis* opined that it does not get hot in the burning Sun and thus is soothing to the oxen while ploughing. Easy workability of *J. regia*, perhaps, guided its use in making tools used in weaving. This has also been noted in other studies [10, 48]. Interestingly, *U. wallichiana* was found to be commonly used for making products that are hollow from inside such as *pedu* and *dhaad*. *Bhangalis* pointed that mature trees of the species are easy for hollowing. Similarly, *Bhangalis* reported that *A. indica* does not easily break on being repeatedly bashed to the ground [41, 60]. This clearly indicates that *Bhangalis* possess knowledge regarding wood and working properties of different species.

In addition to trees, commonly occurring shrubs namley *C. bacillaris*, *D. elegans* and *I. heterantha* were used by the *Bhangalis*. These species are also used by local people in other Himalayan areas [48]. Fibres from herbaceous plant species have also been reported for making mats, ropes and other handicrafts [9]. *Bhangalis* extract fibre from *C. sativa* and use it for making ropes. They mention that fibre from the species is easily extractable and can be woven into various artefacts. Dogan et al [63] also found similar explanations for use of this species. The undercanopy of temperate Himalayan forests is formed by the common hill bamboo *A. falcata*. This alike in other areas [61] was used by the *Bhangalis* for making baskets and other artefacts. Its qualities of strength, light weight and flexibility are well known, which make it a good alternate of timber [64]. Use of residues of agricultural crops namely *H. vulgare* and *T. aestivum* highlight maximization of resource use by the *Bhangalis*. Worshipping *C. deodara*, commonly known as deodar (tree of Gods) and maintenance of traditional conservation practices reflect their views towards conservation and sustainable use of resources [63]. This may be one of the reasons behind every *Bhangali* household having a *mandar* (temple) in their house.

Amongst the PDPs, tools represented the maximum number of utility products used by the *Bhangalis*. This could be attributed to the requirement of diverse implements for carrying out varied daily chores in an efficient and timley manner [10]. It is important to note that practical advancements of humans have been related to innovations in designing tools [65, 66]. Handles of multiple dimensions in different tools represent tweaking for specialized purposes [14]. No doubt, making and assembly of handles by our ancestors using bones is regarded as a revolutionary step in human development [6]. The *Bhangalis* use specially made handles in 17 tools and all of these are made using wood. Wood is used because of its hardness and strength [9]. Fibres from species are used due to their elasticity, ease of extraction, and ability to bear wear and tear [9, 10]. A comparative account of the use of the reported species elsewhere in the Himalaya is presented in Table 4.

### Knowledge comparisons

With reference to sociocultural factors, females, lower age group individuals, and formally educated respondents were found to be less knowledgeable about PDPs. Guimbo et al. [67] also reported that gender and age have strong effects on local knowledge of useful plants. Extraction of resources for PDPs and their making is mostly carried out by males in the present study area and thus their knowledge is expected to be rich and wide. Knowledge enrichment and its differentiation has been reported to be guided by resource access and the social roles performed by different genders [68].

With respect to formally uneducated people having relatively higher knowledge, the same may be due to their direct association with forests and natural resources. They are still involved in activities that are forest oriented. Umair et al. [69] also reported higher traditional knowledge among the non-literate individuals. Similarly, elderly people belonging to fourth age group had more knowledge in comparison to individuals belonging to the first and second age groups (young people). This may be attributed to the temporal advantage that the elder people have. Our results agree with Phillips and Gentry’s [70] proposition that knowledge increases with age. They are in agreement to Muller et al. [39] who showed that gender and age relate to folk knowledge with elder respondents being highly knowledgeable.

**Fig. 5:**
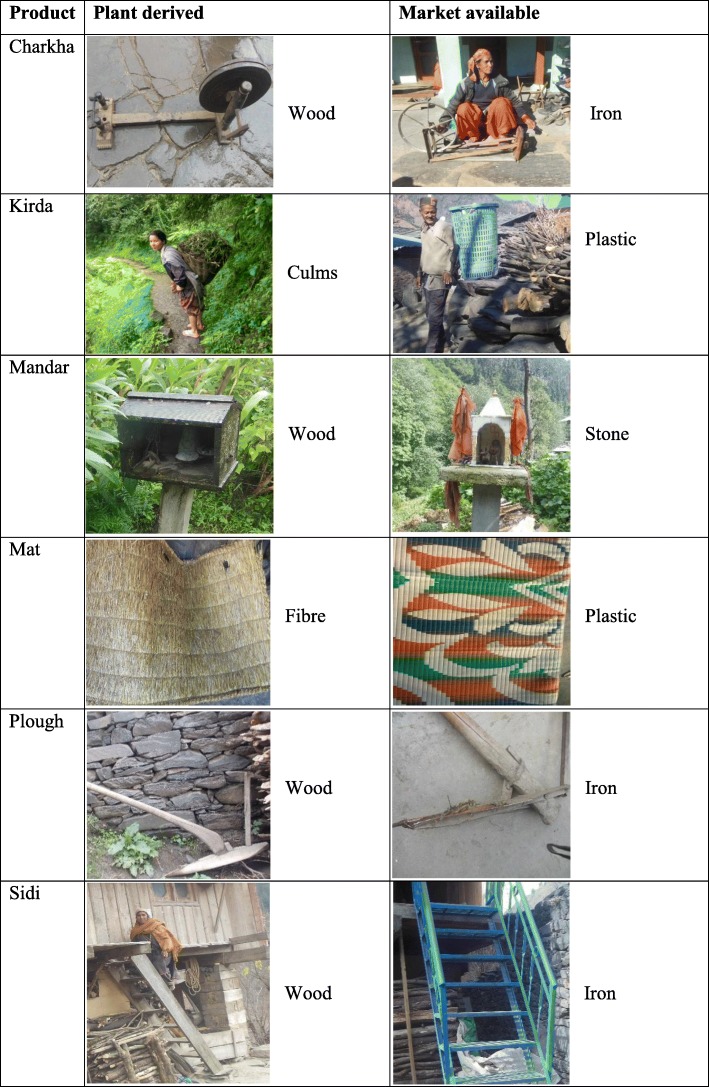
Some examples of market-available alternatives replacing plant-derived products

Unfortunately, recent studies indicate a trend of declining traditional knowlede [71]. *Bhangalis* admit to possessing lesser knowledge in comparison to their forefathers which is validated by the results of age group analyses carried out by us. Across Himalaya, changing lifestyle and market forces have been reported as the prime reasons for this [24, 72]. Similar trends are visible in other parts of the globe [28, 73]. Changing consumption patterns of wild edible plants amongst the *Bhangalis* has also been linked to changing socio-economic conditions [23]. The case with PDPs appears to be no different. It was observed that availability of alternates in the market is resulting in declining use of plants for making products. For many of the daily use products, *Bhangalis* now depend on the market (Fig 5). In the Bhagirathi catchment of western Himalaya also alternatives available in the market have limited the use of plants and the associated knowledge [24, 43]. Thus, it is high time that documentation of plant use knowedge and its prospection is done on a prioity basis. Also, folk knowledge as a subject should be involved in school curriculum such that curosity and its importance are ingrained in the budding period.

## Conclusions

*Bhangalis* use plant-derived products in their day-to-day life for which species commonly occurring in their surroundings are used. *Bhangalis* are aware of the properties and utility of species for making different products. However, this knowledge varies amongst the respondents and is related to gender, age and education. Therefore, comparative studies on the subject become important. Inclusion of folk knowledge as a subject in school curriculum merits a thought.

## Supplementary information

**Additional file 1: Table S1.** General profile of the respondents

## Data Availability

Please contact author for data requests.
