# Supplementary material for: Plant-derived utility products: knowledge comparison across gender, age and education from a tribal landscape of western Himalaya
Source: J Ethnobiol Ethnomed. 2019 Dec 16;15:67. doi: 10.1186/s13002-019-0346-8 (PMC6916028; doi:10.1186/s13002-019-0346-8)
Supplement: Supplementary file 1 — Additional file 1: Table S1. General profile of the respondents [file 13002_2019_346_MOESM1_ESM.docx]

**Table S1** **General profile of the respondents**

| **S. No.** | **Name** | **Age (Years)** | **Gender**  **(M=Male; F=Female)** | **Profession** | **Education**  **(Class passed)** |
| --- | --- | --- | --- | --- | --- |
| 1 | Indira devi* | 47 | F | Zamindaari | 0 |
| 2 | Santi devi* | 51 | F | Zamindaari | 0 |
| 3 | Chudi devi* | 80 | F | Zamindaari | 0 |
| 4 | Ranjeet singh* | 24 | M | Postman/Zamindaari | 12 |
| 5 | Gujri devi* | 50 | F | Zamindaari | 0 |
| 6 | Dyalu devi* | 43 | F | Zamindaari | 0 |
| 7 | Tara chand* | 47 | M | Zamindaari | 2 |
| 8 | Halku ram* | 46 | M | Zamindaari | 0 |
| 9 | Dev raj* | 36 | M | Zamindaari | 12 |
| 10 | Madan lal* | 35 | M | Zamindaari | 4 |
| 11 | Promila* | 23 | F | Tailor/ Zamindaari | 10 |
| 12 | Dhepu* | 70 | F | Zamindaari | 10 |
| 13 | Mahant* | 63 | M | Zamindaari | 0 |
| 14 | Chudu ram* | 80 | M | Zamindaari | 0 |
| 15 | Seru ram* | 82 | M | Zmaindaari | 0 |
| 16 | Mehar singh* | 45 | M | Zamindaari | 0 |
| 17 | Gudi devi* | 35 | F | Zamindaari | 5 |
| 18 | Kishu ram* | 69 | M | Zamindaari | 0 |
| 19 | Nagan* | 64 | F | Zamindaari | 0 |
| 20 | Ram kumar* | 36 | M | Zamindaari | 12 |
| 21 | Kamla* | 17 | F | Zamindaari | 12 |
| 22 | Asha* | 32 | F | Zamindaari | 9 |
| 23 | Champer devi* | 65 | F | Zamindaari | 0 |
| 24 | Doomni * | 85 | F | Zamindaari | 0 |
| 25 | Ghanker* | 65 | F | Zamindaari | 0 |
| 26 | Anarkali* | 54 | F | Zamindaari | 0 |
| 27 | Shyami* | 43 | F | Zamindaari | 0 |
| 28 | Kishu ram* | 65 | M | Zamindaari | 0 |
| 29 | Rachni devi* | 60 | F | Zamindaari | 0 |
| 30 | Chatanki* | 65 | F | Zamindaari | 0 |
| 31 | Sulekha* | 30 | F | Zamindaari | 0 |
| 32 | Bhagat ram* | 65 | M | Zamindaari | 0 |
| 33 | Ghanshyaam* | 64 | M | Zamindaari | 0 |
| 34 | Vipin* | 35 | M | Zamindaari | 0 |
| 35 | Sukhni devi* | 28 | F | Zamindaari | 0 |
| 36 | Sunny* | 26 | M | Zamindaari | 12 |
| 37 | Saran* | 67 | M | Zamindaari | 0 |
| 38 | Dolat ram* | 42 | M | Zamindaari | 12 |
| 39 | Vanu ram* | 68 | M | Zamindaari | 0 |
| 40 | Ambru* | 70 | M | Zamindaari | 0 |
| 41 | Chand ram* | 64 | M | Zamindaar | 5 |
| 42 | Dharmu* | 80 | M | Zamindaari | 0 |
| 43 | Dhani devi* | 55 | F | Zamindaari | 0 |
| 44 | Saju ram* | 60 | M | Zamindaari | 0 |
| 45 | Sunder singh* | 61 | M | Zamindaari | 0 |
| 46 | Hali devi* | 45 | F | Zamindaari | 0 |
| 47 | Makodi* | 80 | F | Zamindaari | 0 |
| 48 | Mala devi* | 34 | F | Zamindaari | 5 |
| 49 | Saraswati* | 65 | F | Zamindaari | 0 |
| 50 | Dugi devi* | 60 | F | Zamindaari | 0 |
| 51 | Mangat ram* | 60 | M | Zamindaari | 0 |
| 52 | Suraj prakash* | 18 | M | Zamindaari | 0 |
| 53 | Khampu ram* | 50 | M | Zamindaari | 0 |
| 54 | Chatanki devi* | 65 | F | Zamindaari | 0 |
| 55 | Chuda ram* | 70 | M | Zamindaari | 0 |
| 56 | Chhikda ram* | 80 | M | Zamindaar | 0 |
| 57 | Guddi devi* | 26 | F | Zamindaari | 0 |
| 58 | Ramesh chand* | 37 | M | Zamindaari | 15 |
| 59 | Meena devi* | 36 | F | Zamindaari | 0 |
| 60 | Pat ram* | 36 | M | Zamindaari | 10 |
| 61 | Ran singh* | 75 | M | Zamindaari | 0 |
| 62 | Snehlata* | 22 | F | Zamindaari | 0 |
| 63 | Gyan chand* | 45 | M | Zamindaari | 8 |
| 64 | Snehlata* | 24 | F | Zamindaari | 12 |
| 65 | Hera lal* | 25 | M | Zamindaari | 8 |
| 66 | Kartik* | 25 | M | Zamindaari | 10 |
| 67 | Raveena* | 21 | F | Patwaari/ Zamindaari | 12 |
| 68 | Rekha* | 27 | F | Zamindaari | 0 |
| 69 | Shyam lal* | 58 | M | Zamindaari | 0 |
| 70 | Janki* | 60 | F | Zamindaari | 0 |
| 71 | Parniti* | 25 | F | Zamindaari | 12 |
| 72 | Balveer singh* | 32 | M | Mechanic/ Zamindaari | 0 |
| 73 | Maheshwar singh* | 27 | M | Driver | 8 |
| 74 | Pragdi* | 70 | F | Zamindaari | 0 |
| 75 | Kishori lal* | 57 | M | Zamindaari | 3 |
| 76 | Seele devi* | 65 | F | Zamindaari | 0 |
| 77 | Bhimkali* | 60 | F | Zamindaar | 0 |
| 78 | Premlata* | 24 | F | Zamindaari | 5 |
| 79 | Vani* | 75 | F | Zamindaari | 0 |
| 80 | Budhi singh* | 43 | M | Zamindaari | 12 |
| 81 | Shanker* | 43 | M | Zamindaari | 9 |
| 82 | Nayan Singh* | 60 | M | Zamindaari | 0 |
| 83 | Bhago Devi* | 80 | F | Zamindaari | 0 |
| 84 | Vanu Ram* | 65 | M | Zamindaari | 5 |
| 85 | Jhingdi* | 70 | F | Zamindaari | 0 |
| 86 | Sukh ram* | 41 | M | Zamindaari | 0 |
| 87 | Achari* | 80 | F | Zamindaari | 0 |
| 88 | Kalidas* | 38 | M | Zamindaari | 4 |
| 89 | Mamta* | 18 | F | Zamindaari | 12 |
| 90 | Billu devi* | 51 | F | Zamindaari | 0 |
| 91 | Dyalu ram* | 51 | M | Zamindaari | 0 |
| 92 | Aasha* | 22 | F | Zamindaari | 12 |
| 93 | Fiti devi* | 55 | F | Zamindaari | 0 |
| 94 | Masti devi* | 60 | F | Zamindaari | 0 |
| 95 | Kaali das* | 26 | M | Zamindaari | 5 |
| 96 | Reeta devi* | 25 | F | Zamindaari | 3 |
| 97 | Neem singh* | 38 | M | Zamindaari | 0 |
| 98 | Kalu ram* | 28 | M | Zamindaari | 10 |
| 99 | Hesi ram* | 59 | M | Zamindaari | 0 |
| 100 | Mohar singh* | 36 | M | Company (job) | 15 |
| 101 | Vikas* | 19 | M | Zamindaari | 12 |
| 102 | Snajeev thakur * | 25 | M | Zamindaari | 17 |
| 103 | Lingri* | 65 | F | Zamindaari | 0 |
| 104 | Kaali devi* | 65 | F | Zamindaari | 0 |
| 105 | Manjeet* | 33 | M | Zamindaari | 10 |
| 106 | Nihaal chand* | 35 | M | Photographer/ Zamindaari | 8 |
| 107 | Kusum* | 29 | F | Zamindaari | 9 |
| 108 | Sushila devi* | 29 | F | Zamindaari | 12 |
| 109 | Sukhni devi* | 28 | F | Zamindaari | 0 |
| 110 | Hemkali* | 31 | F | Zamindaari | 0 |
| 111 | Ramkali* | 53 | F | Zamindaari | 0 |
| 112 | Makholi devi* | 70 | F | Zamindaari | 0 |
| 113 | Bheemkali* | 50 | F | Zamindaari | 0 |
| 114 | Sauni devi* | 80 | F | Zamindaari | 0 |
| 115 | Rangilo devi* | 60 | F | Zamindaari | 0 |
| 116 | Tauri devi* | 75 | F | Zamindaari | 0 |
| 117 | Bidhu* | 70 | M | Zamindaari | 0 |
| 118 | Lalu ram* | 82 | M | Zamindaari | 0 |
| 119 | Radhe shyam* | 62 | M | Zamindaari | 0 |
| 120 | Bresti devi* | 80 | F | Zamindaari | 0 |
| 121 | Jograj * | 38 | M | Zamindaari | 10 |
| 122 | Suman lata* | 35 | F | Anganbaari helper | 10 |
| 123 | Doli* | 80 | F | Zamindaari | 0 |
| 124 | Bahaduri* | 55 | F | Zamindaari | 0 |
| 125 | Ram sharan* | 40 | M | Zamindaari | 0 |
| 126 | Simri devi* | 50 | F | Zamindaari | 0 |
| 127 | Kaltu* | 48 | M | Zamindaari | 2 |
| 128 | Chaangu ram* | 49 | M | PWD worker | 0 |
| 129 | Dhani ram* | 40 | M | Zamindaari | 0 |
| 130 | Kamla* | 40 | F | Zamindaari | 0 |
| 131 | Palku ram* | 65 | M | Zamindaari | 0 |
| 132 | Titli devi* | 80 | F | Zamindaari | 0 |
| 133 | Anarkali* | 65 | F | Zamindaari | 0 |
| 134 | Saraswati* | 31 | F | Zamindaari | 0 |
| 135 | Kali devi* | 85 | F | Zamindaari | 0 |
| 136 | Ramesh chand* | 51 | M | Zamindaari | 0 |
| 137 | Bheemsen* | 48 | M | Zamindaari | 0 |
| 138 | Devraj* | 34 | M | Zamindaari | 4 |
| 139 | Geeta devi* | 32 | F | Zamindaari | 0 |
| 140 | Bhagi devi* | 51 | F | Zamindaari | 0 |
| 141 | Manisha* | 17 | F | Zamindaari | 12 |
| 142 | Rajkumari* | 27 | F | Zamindaari | 0 |
| 143 | Bidhu Ram* | 70 | M | Zamindaari | 0 |
| 144 | Dakar Ram* | 52 | M | Zamindaari | 0 |
| 145 | Subhkaran* | 33 | M | Zamindaari | 5 |
| 146 | Padru* | 70 | M | Zamindaari | 0 |
| 147 | Mahar chand* | 60 | M | Zamindaari | 0 |
| 148 | Seema* | 34 | F | Zamindaari | 0 |
| 149 | Reena* | 26 | F | Zamindaari | 0 |
| 150 | Panjaban devi* | 36 | F | Zamindaari | 0 |
| 151 | Dinesh* | 16 | M | Zamindaari | 12 |
| 152 | Chaitri devi* | 82 | F | Zamindaari | 0 |
| 153 | Surender singh* | 36 | M | Zamindaari | 10 |
| 154 | Dashi devi* | 50 | F | Zamindaari | 0 |
| 155 | Gandhi* | 55 | M | Zamindaari | 0 |
| 156 | Guhiya ram* | 55 | M | Zamindaari | 0 |
| 157 | Famti devi* | 40 | F | Zamindaari | 0 |
| 158 | Riwalsari devi* | 65 | F | Zamindaari | 0 |
| 159 | Sant ram* | 66 | M | Zamindaari | 0 |
| 160 | Rulgad* | 80 | M | Zamindaari | 0 |
| 161 | Mast Ram* | 80 | M | Zamindaari | 0 |
| 162 | Anita Thakur* | 17 | F | Student | 12 |
| 163 | Mangat Ram* | 50 | M | Zamindaari | 0 |
| 164 | Godhma Devi* | 65 | F | Zamindaari | 0 |
| 165 | Pragdu ram* | 58 | M | Zamindaari | 0 |
| 166 | Bhamo devi* | 80 | F | Zamindaari | 0 |
| 167 | Bheemsen* | 54 | M | Zamindaari | 5 |
| 168 | Kali devi* | 55 | F | Zamindaari | 0 |
| 169 | Indira* | 42 | F | Zamindaari | 0 |
| 170 | Prem singh* | 65 | M | Zamindaari | 0 |
| 171 | Budhi singh* | 70 | M | Zamindaari | 0 |
| 172 | Kanori devi* | 45 | F | Zamindaari | 0 |
| 173 | Gyan chand* | 59 | M | Zamindaari | 0 |
| 174 | Mangat Ram* | 65 | M | Zamindaari | 5 |
| 175 | Chuni Lal* | 52 | M | Zamindaari | 0 |
| 176 | Moju ram* | 75 | M | Zamindaari | 0 |
| 177 | Nakhru devi* | 30 | F | Health worker | 0 |
| 178 | Litad ram* | 80 | M | Zamindaari | 0 |
| 179 | Subham* | 16 | M | Student | 10 |
| 180 | Vishavjeet* | 17 | M | Student | 12 |
| 181 | Mehar Singh* | 49 | M | Carpenter | 8 |
| 182 | Roop chand* | 60 | M | Zamindaari | 0 |
| 183 | Nanku* | 63 | M | Zamindaari | 0 |
| 184 | Banto devi* | 42 | F | Zamindaari | 0 |
| 185 | Vaishakhi* | 65 | F | Zamindaari | 0 |
| 186 | Hem Raj* | 19 | M | Zamindaari | 0 |
| 187 | Rammi devi* | 52 | F | Zamindaari | 0 |
| 188 | Rajinder kumar* | 43 | M | Zamindaari | 0 |
| 189 | Jandari* | 85 | F | Zamindaari | 0 |
| 190 | Sunki devi* | 60 | F | Zamindaari | 0 |
| 191 | Kalavati* | 69 | F | Zamindaari | 0 |
| 192 | Vijay kumar* | 30 | M | Zamindaari | 0 |
| 193 | Davinder* | 24 | M | Zamindaari | 0 |
| 194 | Devkali* | 70 | F | Zamindaari | 0 |
| 195 | Chanani devi* | 45 | F | Zamindaari | 0 |
| 196 | Sunil* | 36 | M | Zamindaari | 10 |
| 197 | Ram lal* | 44 | M | Zamindaari | 0 |
| 198 | Saroj* | 22 | F | Zamindaari | 10 |
| 199 | Naro devi* | 40 | F | Zamindaari | 0 |
| 200 | Kamla* | 34 | F | Zamindaari | 0 |
| 201 | Gujri devi* | 55 | F | Zamindaari | 0 |
| 202 | Tara chand* | 53 | M | Zamindaari | 2 |
| 203 | Mehri devi* | 55 | F | Zamindaari | 0 |
| 204 | Sukri devi* | 30 | F | Zamindaari | 0 |
| 205 | Reeta* | 29 | F | Zamindaari | 0 |
| 206 | Amar chand* | 72 | M | Zamindaari | 0 |
| 207 | Kamla devi* | 38 | F | Zamindaari | 2 |
| 208 | Khampi devi* | 51 | F | Zamindaari | 0 |
| 209 | Ramesh chand* | 20 | M | Zamindaari | 0 |
| 210 | Seema devi* | 19 | F | Zamindaari | 12 |
| 211 | Savitri* | 45 | F | Zamindaari | 0 |
| 212 | Santi devi* | 55 | F | Zamindaari | 0 |
| 213 | Roop lal* | 17 | M | Zamindaari | 11 |
| 214 | Hosiar SIngh* | 37 | M | Zamindaari | 3 |
| 215 | AJay* | 25 | M | Zamindaari | 12 |
| 216 | Lali devi* | 82 | F | Zamindaari | 0 |
| 217 | Bharat bhusan* | 45 | M | Zamindaari | 8 |
| 218 | Ravat ram* | 67 | M | Zamindaari | 0 |
| 219 | Ramkali* | 68 | F | Zamindaari | 0 |
| 220 | Ramdhan* | 75 | M | Zamindaari | 5 |
| 221 | Kashmir singh* | 50 | M | PWD worker | 8 |
| 222 | Jogi ram* | 85 | M | Zamindaari | 0 |
| 223 | Dharmi* | 55 | F | Zamindaari | 0 |
| 224 | Sarea* | 31 | F | Zamindaari | 0 |
| 225 | Sunita* | 17 | F | Zamindaari | 8 |
| 226 | Lahuli devi* | 40 | F | Zamindaari | 10 |
| 227 | Sita devi* | 40 | F | Zamindaari | 0 |
| 228 | Vimla devi* | 52 | F | Zamindaari | 0 |
| 229 | Sita ram* | 39 | M | Zamindaari | 5 |
| 230 | Prem chand* | 42 | M | Zamindaari | 7 |
| 231 | Pragdu* | 52 | M | Zamindaari | 2 |
| 232 | Naresh kumar* | 30 | M | Zamindaari | 12 |
| 233 | Bar chand* | 74 | M | Zamindaari | 9 |
| 234 | Ranjeet singh* | 27 | M | Painter | 12 |
| 235 | Maghi devi* | 65 | F | Zamindaari | 0 |
| 236 | Jeev chand* | 60 | M | Zamindaari | 0 |
| 237 | Narayan das* | 70 | M | Zamindaari | 10 |
| 238 | Satya* | 33 | F | Zamindaari | 0 |
| 239 | Sadhu ram* | 67 | M | Zamindaari | 0 |
| 240 | Sillu devi* | 60 | F | Zamindaari | 0 |
| 241 | Rajkumar | 33 | M | Zamindaari | 12 |
| 242 | Ramesh | 27 | M | Zamindaari | 10 |
| 243 | Rani | 23 | F | Zamindaari | 10 |
| 244 | Bishen singh | 80 | M | Zamindaari | 0 |
| 245 | Nanku | 61 | M | Zamindaari | 0 |
| 246 | Reeta | 30 | F | Zamindaari | 10 |
| 247 | Prem singh | 65 | M | Zamindaari | 0 |
| 248 | Sant ram | 50 | M | Zamindaari | 0 |
| 249 | Masti | 92 | F | Zamindaari | 0 |
| 250 | mangat ram | 50 | M | Zamindaari | 0 |
| 251 | joginder | 35 | M | Zamindaari | 9 |
| 252 | Lahuli | 85 | F | Zamindaari | 0 |
| 253 | Damini thakur | 16 | F | Zamindaari | 10 |
| 254 | Kalawati | 80 | F | Zamindaari | 0 |
| 255 | Banto devi | 42 | F | Zamindaari | 0 |
| 256 | Reena devi | 31 | F | Zamindaari | 10 |
| 257 | Gyan chand | 57 | M | Zamindaari | 5 |
| 258 | Shakuntala Devi | 40 | F | Zamindaari | 0 |
| 259 | Dharampal | 32 | M | Zamindaari | 0 |
| 260 | Neelam | 26 | F | Zamindaari | 12 |
| 261 | Sudha | 50 | F | Zamindaari | 0 |
| 262 | Praveen | 27 | M | Zamindaari | 12 |
| 263 | Rancho devi | 50 | F | Zamindaari | 0 |
| 264 | Mohan Das | 35 | M | Zamindaari | 0 |
| 265 | Hoshiyar singh | 35 | M | Zamindaari | 0 |
| 266 | Pritam chand | 60 | M | Zamindaari | 0 |
| 267 | Budhi | 68 | M | Zamindaari | 0 |
| 268 | Prem chand | 60 | M | Zamindaari | 0 |
| 269 | Chood singh | 52 | M | Zamindaari | 5 |
| 270 | Kanta | 30 | F | Zamindaari | 10 |
| 271 | Chhikda ram | 80 | M | Zamindaari | 0 |
| 272 | Makodi devi | 80 | F | Zamindaari | 0 |
| 273 | Bhimkali | 60 | F | Zamindaari | 0 |
| 274 | Bhagvati | 50 | F | Zamindaari | 0 |
| 275 | Visan Das | 40 | M | Zamindaari | 0 |
| 276 | Kishori lal | 54 | M | Zamindaari | 0 |
| 277 | Chood singh | 75 | M | Zamindaari | 0 |
| 278 | Sarswati devi | 60 | F | Zamindaari | 0 |
| 279 | Ratni devi | 45 | F | Zamindaari | 0 |
| 280 | Khampu ram | 50 | M | Zamindaari | 0 |
| 281 | Chamaru ram | 81 | M | Zamindaari | 0 |
| 282 | Guddi devi | 26 | F | Zamindaari | 0 |
| 283 | Shyam lal | 55 | M | Zamindaari | 0 |
| 284 | Kavita | 25 | F | Zamindaari | 12 |
| 285 | Sneh lata | 22 | F | Zamindaari | 12 |
| 286 | Lata | 24 | F | Zamindaari | 12 |
| 287 | Sohni devi | 19 | F | Zamindaari | 10 |
| 288 | Sukhni devi | 38 | F | Zamindaari | 0 |
| 289 | Shanoli | 37 | F | Zamindaari | 0 |
| 290 | Parmiti | 26 | F | Zamindaari | 12 |
| 291 | Ramkali | 55 | F | Zamindaari | 0 |
| 292 | Meenu | 35 | F | Zamindaari | 0 |
| 293 | Bhago devi | 35 | F | Zamindaari | 0 |
| 294 | Ran singh | 45 | M | Zamindaari | 0 |
| 295 | Pankhu devi | 48 | F | Zamindaari | 0 |
| 296 | Sonu | 20 | F | Zamindaari | 10 |
| 297 | Ashu | 20 | F | Zamindaari | 12 |
| 298 | Shauni devi | 28 | F | Zamindaari | 10 |
| 299 | Roshni devi | 30 | F | Zamindaari | 0 |
| 300 | Mohan singh | 48 | M | Zamindaari | 0 |
| 301 | Guddi devi | 45 | F | Zamindaari | 0 |
| 302 | Radhe shyam | 59 | M | Zamindaari | 0 |
| 303 | Bhaggi ram | 64 | M | Zamindaari | 0 |
| 304 | Shauni devi | 80 | F | Zamindaari | 0 |
| 305 | Roshan lal | 30 | M | Zamindaari | 0 |
| 306 | Bhadar singh | 55 | M | Zamindaari | 0 |
| 307 | Lingri devi | 60 | F | Zamindaari | 0 |
| 308 | Bajurgi devi | 80 | F | Zamindaari | 0 |
| 309 | Bhaag singh | 84 | M | Zamindaari | 0 |
| 310 | Mamta | 18 | F | Zamindaari | 10 |
| 311 | Chudi devi | 35 | F | Zamindaari | 0 |
| 312 | Nidhi | 50 | F | Zamindaari | 0 |
| 313 | Banu ram | 65 | M | Zamindaari | 0 |
| 314 | Pandri devi | 55 | F | Zamindaari | 0 |
| 315 | Gopal das | 48 | M | Zamindaari | 0 |
| 316 | Shankar dev | 45 | M | Zamindaari | 0 |
| 317 | Sundri | 48 | F | Zamindaari | 0 |
| 318 | Heera lal | 42 | M | Zamindaari | 0 |
| 319 | Shishu ram | 58 | M | Zamindaari | 0 |
| 320 | Suresh kumar | 32 | M | Zamindaari | 0 |
| 321 | Nanak Chand | 33 | M | Zamindaari | 8 |
| 322 | Baali devi | 27 | F | Zamindaari | 10 |
| 323 | Ranni devi | 60 | F | Zamindaari | 0 |
| 324 | Jhinjdi Devi | 70 | F | Zamindaari | 0 |
| 325 | Nigri devi | 60 | F | Zamindaari | 0 |
| 326 | Sukh dev | 27 | M | Zamindaari | 12 |
| 327 | Jai krishan | 30 | M | Zamindaari | 10 |
| 328 | Hesi ram | 56 | M | Zamindaari | 0 |
| 329 | Sauni devi | 80 | F | Zamindaari | 0 |
| 330 | Bhag singh | 84 | M | Zamindaari | 0 |
| 331 | Ram lal | 45 | M | Zamindaari | 0 |
| 332 | Suri das | 38 | M | Zamindaari | 0 |
| 333 | Rani devi | 38 | F | Zamindaari | 0 |
| 334 | Bhagwan das | 55 | M | Zamindaari | 0 |
| 335 | Chhaji devi | 60 | F | Zamindaari | 0 |
| 336 | Manju | 33 | F | Zamindaari | 10 |
| 337 | Bhagat ram | 43 | M | Zamindaari | 0 |
| 338 | Sharvan ram | 42 | M | Zamindaari | 5 |
| 339 | Shauju ram | 60 | M | Zamindaari | 0 |
| 340 | Charanjeet | 47 | M | Zamindaari | 0 |
| 341 | Kamla | 40 | F | Zamindaari | 0 |
| 342 | Sansar chand | 50 | M | Zamindaari | 0 |
| 343 | Magu ram | 70 | M | Zamindaari | 0 |
| 344 | Durgi devi | 60 | F | Zamindaari | 0 |
| 345 | Harsh | 33 | M | Zamindaari | 12 |
| 346 | Jehru ram | 85 | M | Zamindaari | 0 |
| 347 | Kishi devi | 55 | F | Zamindaari | 0 |
| 348 | Vimla devi | 50 | F | Zamindaari | 0 |
| 349 | Bindu ram | 50 | M | Zamindaari | 0 |
| 350 | Vansuri devi | 60 | F | Zamindaari | 0 |
| 351 | Hoshiyar singh | 50 | M | Zamindaari | 0 |
| 352 | Guddi devi | 47 | F | Zamindaari | 0 |
| 353 | Dasori ram | 86 | M | Zamindaari | 0 |
| 354 | Satya devi | 45 | F | Zamindaari | 0 |
| 355 | Prem chand | 48 | M | Zamindaari | 0 |
| 356 | Tej ram | 60 | M | Zamindaari | 0 |
| 357 | Prem kali | 40 | F | Zamindaari | 0 |
| 358 | Kagadi devi | 50 | F | Zamindaari | 0 |
| 359 | Chatar singh | 60 | M | Zamindaari | 0 |
| 360 | Gori devi | 47 | F | Zamindaari | 0 |
| 361 | Roop lal | 65 | M | Zamindaari | 0 |
| 362 | Kanya | 50 | F | Zamindaari | 0 |
| 363 | Kartar | 68 | M | Zamindaari | 0 |
| 364 | Sukh ram | 42 | M | Zamindaari | 0 |
| 365 | Nakhru | 30 | F | Zamindaari | 10 |
| 366 | Bhuri singh | 61 | M | Zamindaari | 0 |
| 367 | Janki devi | 75 | F | Zamindaari | 0 |
| 368 | Budhi devi | 29 | F | Zamindaari | 0 |
| 369 | Jandru | 75 | M | Zamindaari | 0 |
| 370 | Asha | 30 | F | Zamindaari | 12 |
| 371 | Halku ram | 45 | M | Zamindaari | 7 |
| 372 | Jhagdu | 70 | M | Zamindaari | 0 |
| 373 | Sunil kumar | 35 | M | Zamindaari | 0 |
| 374 | Sauni devi | 90 | F | Zamindaari | 0 |
| 375 | Rajkumar | 29 | M | Zamindaari | 12 |
| 376 | Khudu ram | 64 | M | Zamindaari | 0 |
| 377 | Pandri devi | 68 | F | Zamindaari | 0 |
| 378 | Rajkumar | 33 | M | Zamindaari | 10 |
| 379 | Nanak chand | 60 | M | Zamindaari | 0 |
| 380 | Hiri devi | 36 | F | Zamindaari | 10 |
| 381 | Mashadu | 72 | M | Zamindaari | 0 |
| 382 | Saran das | 58 | M | Zamindaari | 0 |
| 383 | Maagga ram | 38 | M | Zamindaari | 0 |
| 384 | Sant ram | 48 | M | Zamindaari | 0 |
| 385 | Beerbal | 43 | M | Zamindaari | 0 |
| 386 | Anita devi | 30 | F | Zamindaari | 12 |
| 387 | Jalahi devi | 75 | F | Zamindaari | 0 |
| 388 | Sushma | 31 | F | Zamindaari | 12 |
| 389 | Mahant Ram | 63 | M | Zamindaari | 0 |
| 390 | Aanand lal | 35 | M | Zamindaari | 15 |
| 391 | Guddi devi | 33 | F | Zamindaari | 12 |
| 392 | Rasal singh | 60 | M | Zamindaari | 0 |
| 393 | Joginder singh | 41 | M | Zamindaari | 0 |
| 394 | Chamari | 75 | F | Zamindaari | 0 |
| 395 | Bhadri devi | 46 | F | Zamindaari | 0 |
| 396 | Ragan devi | 85 | F | Zamindaari | 0 |
| 397 | Indira Devi | 34 | F | Zamindaari | 10 |
| 398 | Pragdi devi | 55 | F | Zamindaari | 0 |
| 399 | Roshan lal | 48 | M | Zamindaari | 0 |
| 400 | Sumna devi | 20 | F | Zamindaari | 12 |
| 401 | Shyam lal | 44 | M | Zamindaari | 10 |
| 402 | Shukri devi | 38 | F | Zamindaari | 5 |
| 403 | Begi devi | 45 | F | Zamindaari | 0 |
| 404 | Devkali | 38 | F | Zamindaari | 0 |
| 405 | Partap chand | 35 | M | Zamindaari | 12 |
| 406 | Durga | 25 | F | Zamindaari | 10 |
| 407 | Rampal | 64 | M | Zamindaari | 0 |
| 408 | Fula devi | 34 | F | Zamindaari | 12 |
| 409 | Sumna | 35 | F | Zamindaari | 12 |
| 410 | Reshmu | 75 | F | Zamindaari | 0 |
| 411 | Survdyal | 37 | M | Zamindaari | 12 |
| 412 | San ram | 50 | M | Zamindaari | 0 |
| 413 | Chood singh | 52 | M | Zamindaari | 0 |
| 414 | Sumna | 40 | F | Zamindaari | 0 |
| 415 | Kannori devi | 26 | F | Zamindaari | 12 |
| 416 | Nandni devi | 37 | F | Zamindaari | 0 |
| 417 | Ankush | 17 | M | Zamindaari | 10 |
| 418 | Chatri devi | 80 | F | Zamindaari | 0 |
| 419 | Shauni devi | 55 | F | Zamindaari | 0 |
| 420 | Roshan lal | 35 | M | Zamindaari | 10 |

*Respondents selected for age and gender analyses, Zamindaari=farming
